# Supplementary figures and images for: Chikungunya Outbreak in the Republic of the Congo, 2019—Epidemiological, Virological and Entomological Findings of a South-North Multidisciplinary Taskforce Investigation
Source: Viruses. 2020 Sep 13;12(9):1020. doi: 10.3390/v12091020 (PMC7551106; doi:10.3390/v12091020)

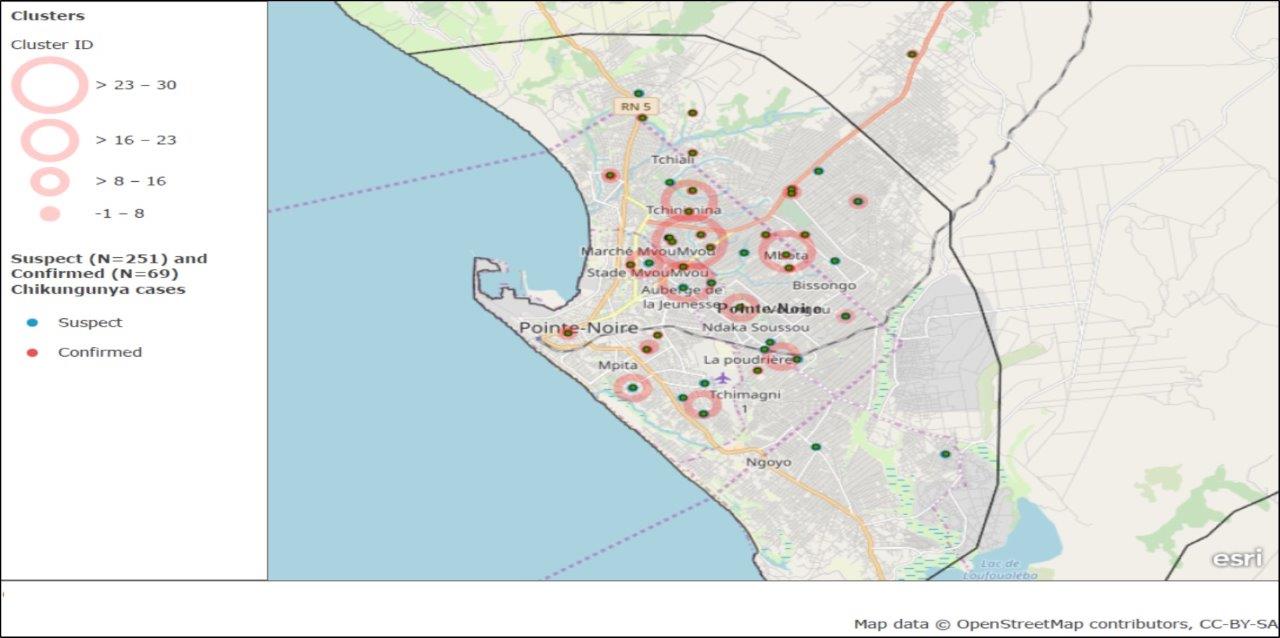

Supplement: Supplementary file 1 [file viruses-12-01020-s001.zip › Supplementary material/Fig. S1_ Geolocalisation.jpg]
